# Predictors of cognitive and physical decline: Results from the Health Aging and Body Composition Study

**DOI:** 10.3389/fnagi.2023.1122421

**Published:** 2023-02-20

**Authors:** Elizabeth P. Handing, Kathleen M. Hayden, Xiaoyan Iris Leng, Stephen B. Kritchevsky

**Affiliations:** ^1^Department of Human Development and Family Studies, Colorado State University, Fort Collins, CO, United States; ^2^Division of Public Health Sciences, Wake Forest School of Medicine, Winston-Salem, NC, United States; ^3^Department of Biostatistics and Data Sciences, Wake Forest School of Medicine, Winston-Salem, NC, United States; ^4^Department of Internal Medicine, Section on Gerontology and Geriatric Medicine, Sticht Center for Healthy Aging and Alzheimer’s Prevention, Wake Forest School of Medicine, Winston-Salem, NC, United States

**Keywords:** cognitive decline, physical decline, aging, risk factors, depressive symptoms, dual decline

## Abstract

**Background:**

Risk factors for cognitive decline and physical decline have been studied independently, however older adults might experience decline in both areas i.e., dual decline. Risk factors associated with dual decline are largely unknown and have significant implications on health outcomes. The aim of this study is to explore risk factors associated with dual decline.

**Methods:**

Using data from the Health, Aging and Body Composition (Health ABC) study, a longitudinal prospective cohort study, we examined trajectories of decline based on repeated measures of the Modified Mini-Mental State Exam (3MSE) and the Short Physical Performance Battery (SPPB) across 6 years (*n*=1,552). We calculated four mutually exclusive trajectories of decline and explored predictors of decline: cognitive decline (*n* = 306) = lowest quartile of slope on the 3MSE or 1.5 SD below mean at baseline, physical decline (*n* = 231) = lowest quartile of slope on the SPPB or 1.5 SD below mean at baseline, dual decline (*n* = 110) = lowest quartile in both measures or 1.5 SD below mean in both measures at baseline. Individuals who did not meet criteria for one of the decline groups were classified as the reference group. (*n*= 905).

**Results:**

Multinomial logistic regression tested the association of 17 baseline risk factors with decline. Odds of dual decline where significantly higher for individuals at baseline with depressive symptoms (CES-D >16) (Odds Ratio (OR)=2.49, 95% Confidence Interval (CI): 1.05-6.29), *ApoE-ε4* carrier (OR= 2.09, 95% CI: 1.06-1.95), or if individuals had lost 5+lbs in past year (OR=1.79, 95% CI: 1.13-2.84). Odds were significantly lower for individuals with a higher score on the Digit Symbol Substitution Test per standard deviation (OR per SD: 0.47, 95% CI 0.36-0.62) and faster 400-meter gait (OR per SD= 0.49, 95% CI: 0.37-0.64).

**Conclusion:**

Among predictors, depressive symptoms at baseline significantly increased the odds of developing dual decline but was not associated with decline in the exclusively cognitive or physical decline groups. *APOE*-ε4 status increased the odds for cognitive decline and dual decline but not physical decline. More research on dual decline is needed because this group represents a high risk, vulnerable subset of older adults.

## 1. Introduction

Declines in cognitive and physical function are major concerns for older adults, and can result in loss of independence, higher health care utilization, and increased risk for dementia (Verghese et al., 2002; Hardy et al., 2011). The research community has commonly viewed these two abilities as independent trajectories, although emerging research is beginning to show a consensus that cognitive abilities and physical abilities are correlated, dynamic, and bidirectional (Tabbarah et al., 2002; Atkinson et al., 2007, 2010; Fitzpatrick et al., 2007; Inzitari et al., 2007; Rosano et al., 2008; Soumare et al., 2009; Watson et al., 2010; Mielke et al., 2013; Gothe et al., 2014; Krall et al., 2014; Best et al., 2016; Finkel et al., 2016; Montero-Odasso et al., 2019; Okley and Ian, 2020). A meta-analysis by Clouston and colleagues (Clouston et al., 2013) found evidence from 36 longitudinal studies consistently showing a correlation between physical function and cognitive function, although the strength of the association varied depending on assessment type. For example, grip strength was associated with changes in global cognition, while walking speed was correlated with changes in fluid cognition. Few studies have modeled changes in cognitive function and physical function together as a dual process longitudinally, i.e., dual decline. In prior studies examining combined decline (Montero-Odasso et al., 2020; Tian et al., 2020; Collyer et al., 2022) the authors primarily investigated dual decline as a predictor for dementia, which all three studies found significant associations. Additionally, each of those studies used scores from gait speed only and cognition/memory to define dual decline. In the current study, we seek to define dual decline by using a variety of physical function tests that represent different domains of function including balance, sit to stand, and walk speed.

What has not been well characterized are if there are certain predictors that predispose an individual for dual decline. The first study to examine predictors of dual decline was published in 2005 and identified smoking and low hemoglobin as significant predictors (Atkinson et al., 2005), albeit the sample only included 522 older women. Since then, little work has been conducted on risk factors of decline and thus a gap in the literature exists.

The purpose of this study is to (a) define four mutually exclusive groups (dual decline, cognitive decline only, physical decline only, and a reference group) and (b) explore predictors that may have a particularly strong association with dual decline. Determining predictors and modeling dual decline may help in early identification of a high-risk group of older adults and potentially develop interventions in order to prevent poor health outcomes in the future.

## 2. Materials and methods

Our study includes information from over 1,500 older adults from the Health, Aging and Body Composition (Health ABC) study, a longitudinal prospective cohort study of well-functioning, community dwelling older adults with a comprehensive examination of physical function, cognitive function, health data, and biomarkers. Health ABC recruited 3,075 men and women aged 70–79 years from a random sample of White and Black Medicare eligible residents in the Pittsburgh, PA, and Memphis, TN, metropolitan areas between April 1997 and June 1998 (51.5% female, 41.7% African American). Participants were eligible if they reported no difficulty walking ¼ mile, climbing 10 steps, or performing basic activities of daily living.

### 2.1. Subject selection

For this project, we examined previously collected data across 6 years (1997/1998–2002/2003). We considered baseline to be inclusive of data through the 36-month visit (to include certain biomarkers not collected at month 0). All participants were free of mobility and cognitive impairments at baseline per self-report. Trajectories of decline were evaluated from three timepoints across 6 years. Participants who completed the Modified Mini-Mental State Exam (3MSE) and Short Physical Performance Battery (SPPB) at baseline with at least one successive measure after baseline were included in the analysis to calculate the slope. The SPPB was collected at the 0-, 48-, and 72-month follow-up visits and the 3MSE was collected at 0-, 36-, and 60-month follow-up visits.

Participants were excluded if they had a previous stroke (*n* = 88), Parkinson’s Disease (*n* = 21), or died before the 72-month visit (*n* = 384). Participants with only one measure of 3MSE or SPPB were excluded from analyses (*n* = 329). Complete case analysis was used and participants with missing baseline variables were excluded (*n* = 701). The final sample size was 1,552.

### 2.2. Risk factors at baseline

Selection of risk factors in this study were based upon previous research in this area (Atkinson et al., 2005) and we hypothesized that poor metabolic health (i.e., diabetes, hypertension, current smoker, alcohol drinker, high body mass index, and low hemoglobin) would be a particularly potent set of risk factors for those with dual decline.

### 2.3. Demographic variables

Demographic information and health questions were collected from self-report and included: age, sex, years of education completed, marital status, race (black, white), weight history, and self-reported health (poor, fair, good, very good, excellent). Participants were asked about smoking (are you a current smoker), and alcohol intake (do you currently drink alcohol, and how much per day). Participants were asked if they had fallen in the past 12 months (dichotomized to ≤ 1 time or 2 + times) and/ or hospitalizations in the past 12 months (Yes or No). Disease status for diabetes and hypertension was ascertained from the question at baseline, “Has a doctor ever told you that you have….” Depressive symptoms were assessed using the Center for Epidemiologic Studies Depression Scale (CES-D 20; Radloff, 1977). A score of 16 is the screening cut-off for risk of clinical depression.

### 2.4. Functional variables

Objective measures of functioning were also collected at an in-person clinic visit. Body mass index was calculated as weight/height (m)^2^ (Fitzpatrick et al., 2007). Lung function was measured as the percent predicted forced expiratory volume in 1 s (pFEV1). A value of less than 80 was used to indicate poor lung function. Hand grip strength was calculated as the average of two trials in the right hand using an adjustable grip strength dynamometer. Grip strength was adjusted for gender and body weight. Participants also completed a 400-m walk at baseline. Executive function was measured using the Digit Symbol Substitution Test (DSST; Wechsler, 1997).

### 2.5. Biomarker variables

Blood samples were collected *via* a venipuncture during an in-person baseline assessment. The biomarkers chosen for this project included: total cholesterol (mg/dL), hemoglobin (g/dL), serum albumin (g/dL), and serum vitamin D (25-hydroxyvitamin D; ng/mL) deficient (< 20 ng/ml), and at least one Apolipoprotein ε4 allele (*APOE*-ε4). These biomarkers were selected based upon previous studies (Atkinson et al., 2005, 2007) and are known to influence physical function and cognitive function. All biomarkers were collected at month 0 with the exception of hemoglobin (values were from the 36-month visit because it was not collected at month 0, and serum vitamin D is from the 24-month visit).

### 2.6. Outcome variables

Physical function was measured using the SPPB (Guralnik et al., 1994). The SPPB is composed of three physical function domains: a balance test, an 8-m walk, and a timed chair sit to stand. Scores from each domain were summed to create a composite score which ranges from 0 to 12 with higher scores indicating better performance. Cognitive function was measured using the 3MSE (Teng and Chui, 1987). The 3MSE includes tests of orientation, registration, attention, calculation, recall, and visual–spatial skills. Scores can range from 0 to 100 points, with higher scores indicating better performance.

Four trajectory groups were defined by a decline in the slope across 6 years (0–72 months) using repeated measures from participant-specific slopes of 3MSE and SPPB scores. Those with a predicted slope in the lowest quartile or 1.5 SD below the mean at baseline, exclusively in cognition or physical function were classified as “cognitive decline” only or “physical decline” only. Those who met the same criteria for both cognitive and physical decline were classified as “dual decline.” Individuals who did not meet criteria for one of the decline groups were classified as the reference group.

### 2.7. Analytic approach

The four trajectory groups were defined based upon participant-specific slopes of 3MSE and SPPB scores from 0 to 72 months. Cognitive decline = lowest quartile of 3MSE slope or 1.5 SD below the mean at month 0, physical decline = lowest quartile of SPPB or 1.5 SD below the mean at month 0, and dual decline = lowest slope quartiles of 3MSE and SPPB or 1.5 SD below the mean in both domains at month 0. Participants who did not meet criteria for one of the decline groups were categorized as the reference group. Descriptive statistics were used to describe group characteristics with baseline predictors (Chi-square for proportions, and ANOVA for continuous variables). Next, a risk profile was constructed to identify which variables from baseline were associated with membership of each prospective decline category. Multinomial logistic regression was performed to model decline category with significant baseline variables as predictors. Hemoglobin g/dL, serum albumin g/dL, grip strength kg, DSST, and 400 m walk m/s were converted to z-scores for ease of interpretation. Odds Ratios and 95% confidence intervals (95% CI) are presented. All analyses were conducted using SAS 9.4.

## 3. Results

Characteristics of the four trajectory groups are presented in Table 1. The cognitive decline group (*n* = 306) had an average 3MSE score of 88.8 at baseline and decreased on average 1.1 points per year. The physical decline group (*n* = 231) had an average SPPB score of 10.2 at baseline decreased by 0.55 points per year. The dual decline group (*n* = 110) had an average 3MSE score of 89 and SPPB score of 10.0 at baseline and decreased by 2.40 points on the 3MSE and 0.76 points on the SPPB per year. The dual decline group was significantly older, included more women and those who were less educated, black, and those less likely to be married, and more likely to self-report having lost 5 or more pounds in the past year. They also had more depressive symptoms, poorer self-rated health, and were less likely to be a current alcohol drinker compared to the reference group. The dual decline group also had significantly lower grip strength, lower hemoglobin (g/dL), albumin (g/dL), were more likely to be deficient in serum vitamin D (25-hydroxyvitamin D; < 20 ng/ml ng/mL), and to have at least one *APOE*-ε4 allele.

**Table 1:** Descriptive characteristics at baseline across four groups of decline, data from the Health, Aging and Body Composition (Health ABC) Study (*n* = 1,552).

	Reference group (*n* = 905)	Cognitive decline (*n* = 306)	Physical decline (*n* = 231)	Dual decline (*n* = 110)	Value of *p*
*Baseline characteristics:*					
Health					
Age, mean (SD)	73.1 (2.7)	73.6 (3.0)	73.7 (2.9)	74.4 (3.0)	<0.001
Women, *n* (%)	428 (47.3)	148 (48.3)	141 (61.0)	67 (60.9)	<0.001
Education, *n* (%)					
≤ High school	399 (44.1)	202 (66.0)	116 (50.2)	77 (70.0)	<0.001
Race, *n* (%)					
White	677 (74.8)	160 (52.3)	164 (71.0)	55 (50.0)	<0.001
Black	228 (25.2)	146 (47.7)	67 (29.0)	55 (50.0)	
BMI, mean (SD)	26.9 (4.1)	27.2 (4.3)	27.7 (5.1)	27.4 (5.6)	0.047
Not Married, *n* (%)	387 (42.7)	133 (43.5)	123 (53.3)	61 (55.5)	0.004
Weight history					
Gained 5 + lbs. in past year	273 (31.0)	103 (34.6)	78 (34.2)	31 (29.0)	0.523
Lost 5 + lbs. in past year	260 (28.7)	103 (33.7)	85 (36.8)	47 (42.7)	0.004
Self-rated health, poor, *n* (%)	66 (7.3)	59 (19.3)	25 (10.8)	23 (20.9)	<0.001
Lifestyle *n*, %					
Current smoker	61 (6.7)	25 (8.2)	17 (7.4)	11 (10.0)	0.587
Smoked 100 + cigarettes	501 (55.4)	152 (49.7)	120 (52.0)	57 (51.8)	0.649
Current alcohol drinker	516 (57.0)	135 (44.1)	124 (53.7)	40 (36.4)	<0.001
Depressive symptoms, %	24 (2.7)	8 (2.6)	6 (2.6)	11 (10.0)	<0.001
Grip Strength (kg), mean (SE)	31.6 (0.3)	32.4 (0.6)	28.3 (0.7)	28.8 (0.9)	<0.001
Walk > 150 min a week	297 (33.0)	85 (28.2)	74 (32.0)	36 (32.7)	0.138
PFEV1 < 80%	196 (21.7)	73 (23.9)	53 (22.9)	22 (20.0)	0.796
Fallen in past year	202 (22.4)	54 (17.7)	50 (21.8)	23 (21.1)	0.386
Two or more times	42 (4.7)	12 (3.9)	17 (7.4)	7 (6.4)	0.250
Been hospitalized in past year	102 (11.3)	31 (10.1)	26 (11.3)	13 (11.8)	0.944
Two or more times	16 (1.8)	4 (1.3)	3 (1.3)	1 (0.9)	0.851
Biomarkers, mean (SD)					
Total cholesterol (mg/dL)	204.1 (38.3)	201.5 (36.7)	200.2 (36.0)	205.1 (35.3)	0.410
Hemoglobin (g/dL)	13.8 (1.2)	13.6 (1.4)	13.6 (1.3)	13.3 (1.3)	<0.001
Serum albumin (g/dL)	4.0 (0.3)	3.9 (0.3)	4.0 (0.3)	3.9 (0.3)	<0.001
Serum Vitamin D (25-hydroxyvitamin D) (ng/mL)	27.9 (10.1)	25.2 (10.3)	26.2 (11.7)	26.2 (10.0)	<0.001
Deficient (< 20 ng/ml), *n* (%)	212 (23.4)	100 (32.7)	77 (33.3)	34 (30.0)	0.001
ApoE-4 +	219 (24.2)	101 (33.0)	46 (19.9)	45 (40.9)	<0.001
Chronic disease, *n* (%)					
Hypertension	404 (44.6)	136 (44.4)	120 (52.0)	58 (52.7)	0.102
Diabetes	96 (10.6)	36 (11.8)	31 (13.4)	18 (16.4)	0.258
Other, mean (SD)					
3MSE	93.0 (4.8)	88.8 (9.4)	93.0 (4.8)	89.0 (7.9)	<0.001
SPPB	10.5 (1.1)	10.3 (1.2)	10.2 (1.8)	10.0 (1.6)	0.001
DSST	42.1 (11.7)	32.9 (13.2)	39.3 (13.0)	30.4 (12.7)	<0.001
400 m gait speed, m/s (SD)	1.3 (0.2)	1.2 (0.2)	1.2 (0.2)	1.1 (0.2)	<0.001

BMI = body mass index, PFEV1 = predicted forced expiratory volume in 1 s, Depressive symptoms = score of > 16 on the Center for Epidemiologic Studies Depression Scale (CES-D-20), ApoE-4 + = Apolipoprotein e4 +, 3MSE = Modified Mini Mental Status Exam, SPPB = Short Physical Performance Battery, DSST = Digit Symbol Substitution Test.

**Figure 1:**
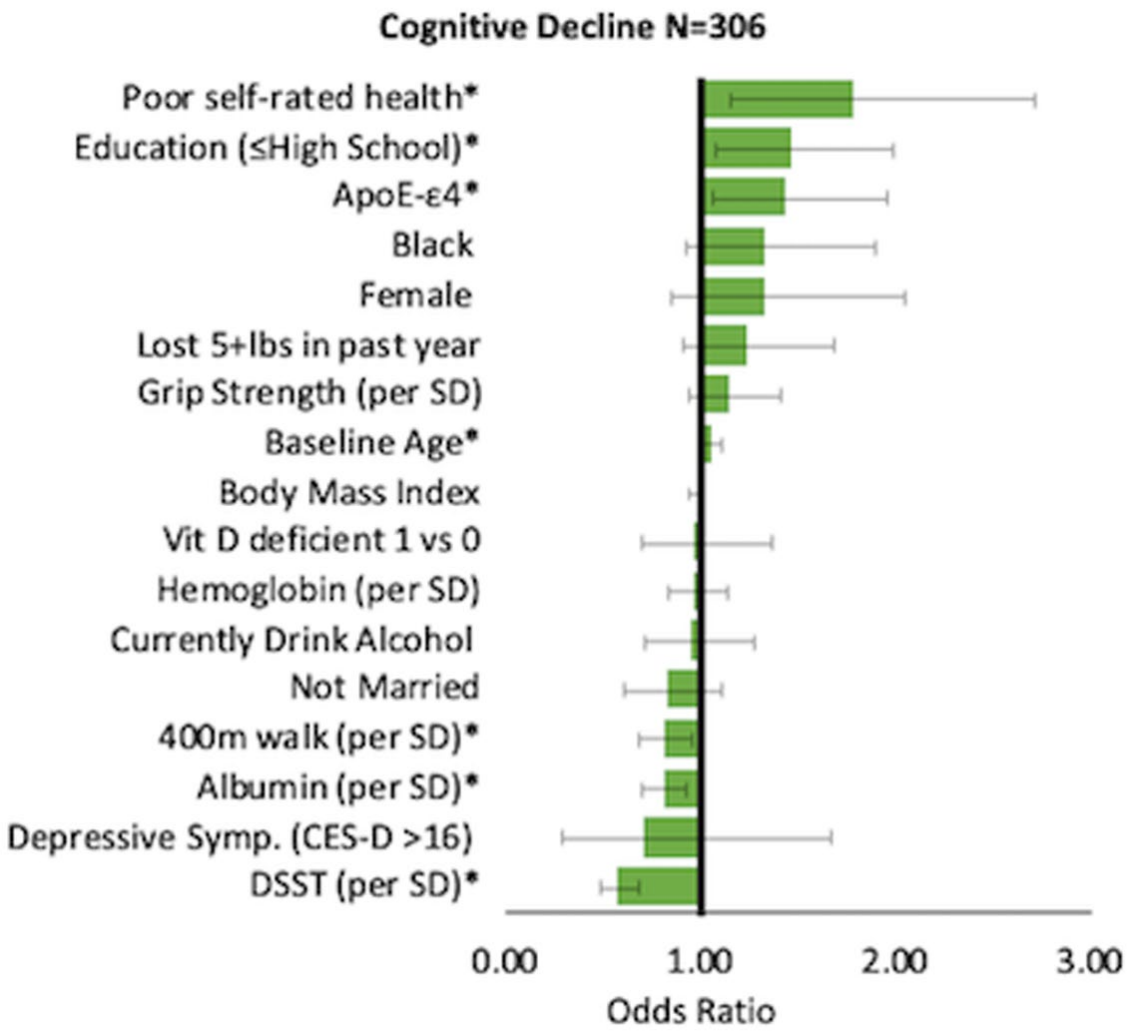
Correlates of cognitive decline. Odds Ratio [95% CI] compared to the reference group.

**Figure 2:**
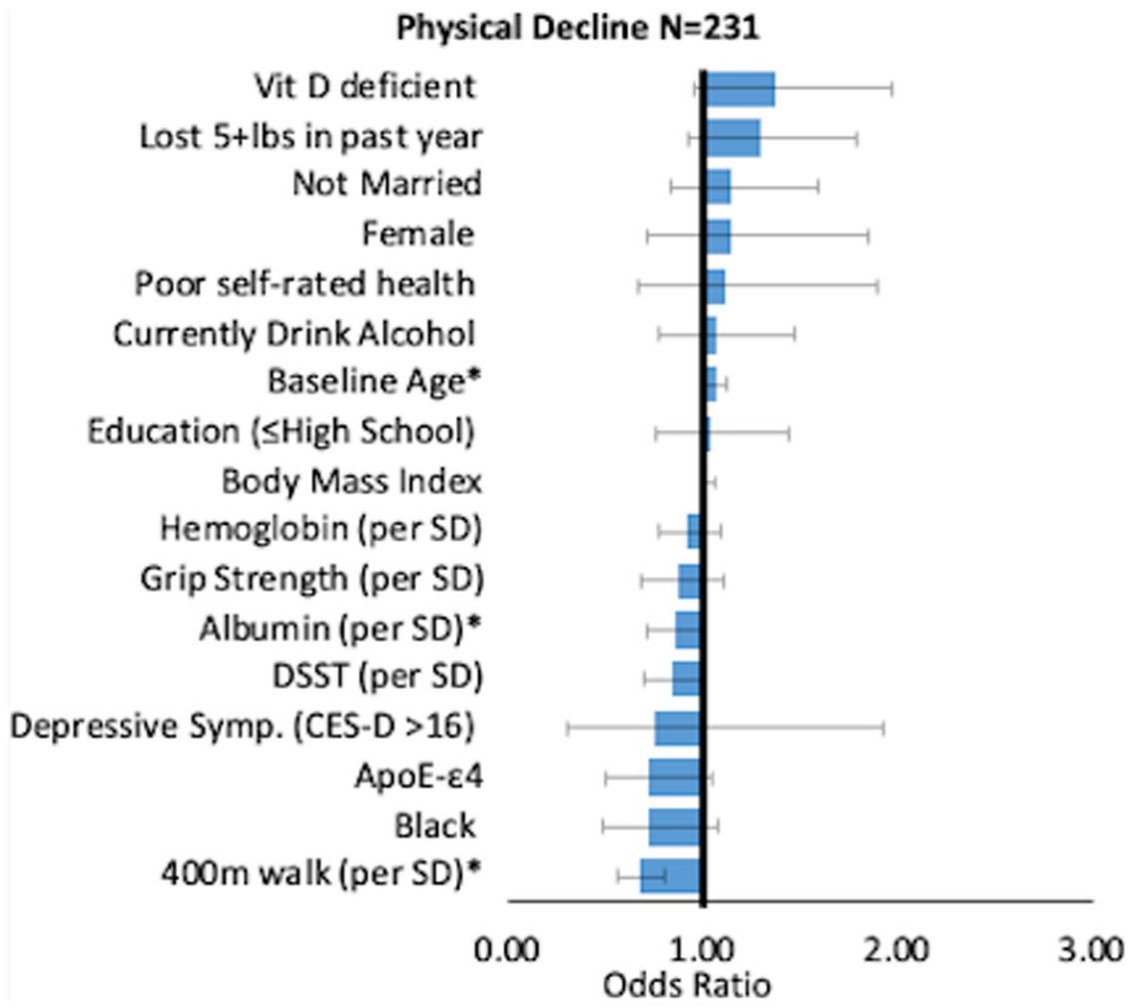
Correlates of physical decline. Odds Ratio [95% CI] compared to the reference group.

When significant variables from baseline were entered into a multinomial logistic regression model, significant risk factors of cognitive decline were higher age (OR = 1.05, 95% CI: 1.00–1.11), low education (≤ high school; OR = 1.46, 95% CI: 1.07–1.98), poor self-rated health (OR = 1.78, 95% CI: 1.16–2.71) and *APOE*-ε4 (OR = 1.44, 95% CI: 1.06–1.95). Higher serum albumin (OR per standard deviation (SD) = 0.81, 95% CI: 0.95–0.98), higher DSST score (OR per SD: 0.57, 95% CI: 0.48–0.68), and faster 400 m walk (OR per SD 0.81, 95% CI: 0.68–0.96) were significantly associated with lower odds of cognitive decline (Figure 1). Physical decline predictors are depicted in Figure 2. Higher age (OR = 1.07, 95% CI: 1.01–1.13) increased the odds, while serum albumin (OR per SD = 0.85, 95% CI 0.72–0.99) and 400 m (OR per SD = 0.67, 95% CI: 0.56–0.81) decreased the odds.

**Figure 3:**
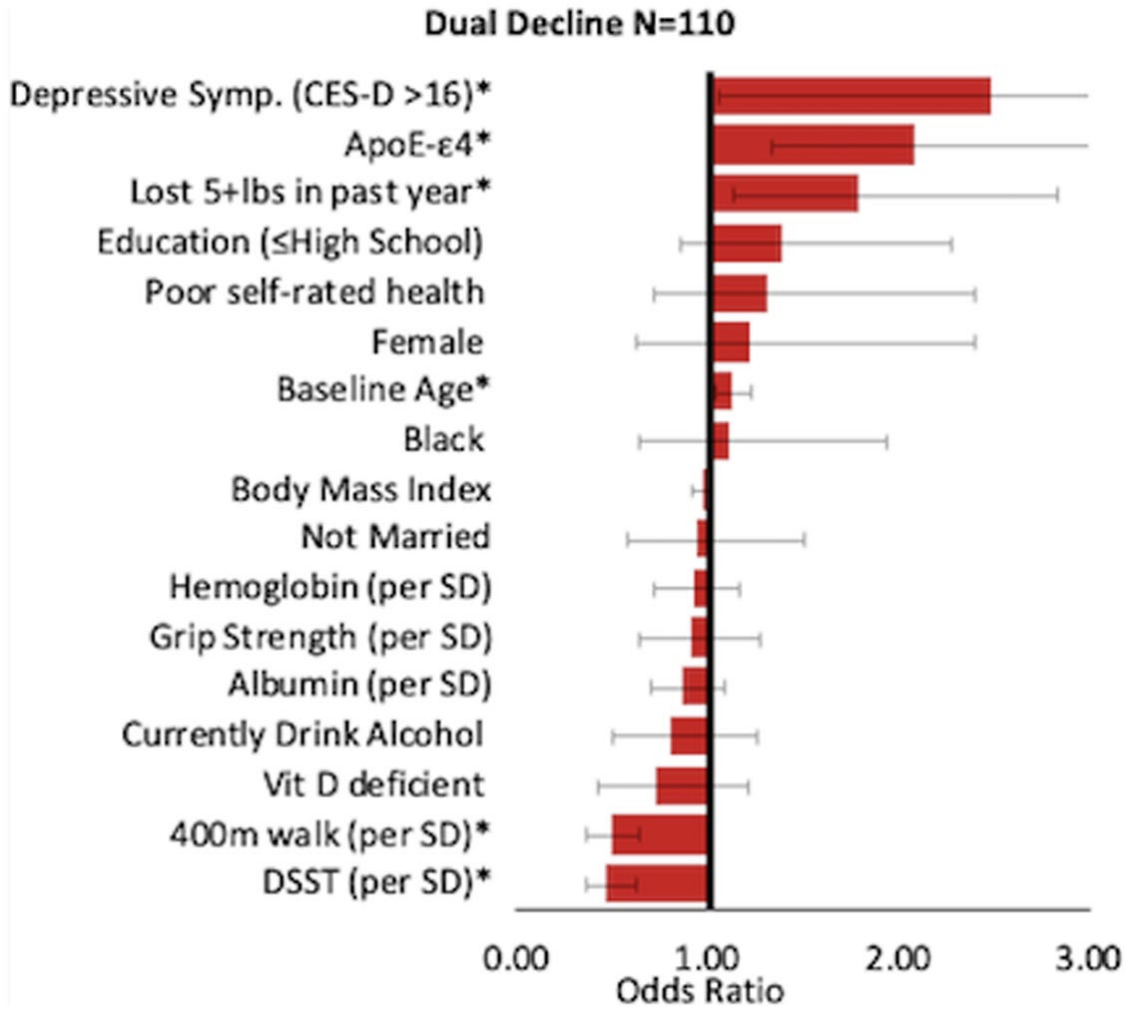
Correlates of dual decline. Odds Ratio [95% CI] compared to the reference group.

Risk factors for dual decline included: age (OR = 1.13, 95% CI: 1.04–1.22), lost 5 + lbs. in past year (OR = 1.79, 95% CI: 1.13–2.84), depressive symptoms (OR = 2.49, 95% CI: 1.05–5.91), and *APOE*-ε4 (OR = 2.09, 95% CI: 1.33–3.28). Higher scores on the DSST and faster 400 m walking speed were significantly related to lower odds of dual decline (OR per SD = 0.47, 95% CI: 0.36–0.62; OR per SD = 0.49, 95% CI 0.37–0.64), respectively (Figure 3). OR and 95% CIs for all groups are presented in Table 2.

**Table 2:** Odds ratios [95% CI] with all significant predictors from regression analyses with comparison to the reference group.

	Cognitive decline (*n* = 306)			Physical decline (*n* = 231)			Dual decline (*n* = 110)		
	OR	95% CI		OR	95% CI		OR	95% CI	
Health									
Age	**1.05**	1.00	1.11	**1.07**	1.01	1.13	**1.13**	1.04	1.22
Female	1.32	0.85	2.05	1.14	0.71	1.85	1.22	0.62	2.40
Education (≤ High School)	**1.46**	1.07	1.98	1.04	0.76	1.44	1.39	0.85	2.27
Race (Black)	1.33	0.93	1.90	0.72	0.48	1.08	1.11	0.64	1.94
Body Mass Index	0.98	0.94	1.01	1.02	0.98	1.06	0.97	0.92	1.01
Not married	0.82	0.60	1.11	1.15	0.84	1.59	0.94	0.58	1.51
Lost 5 + lbs. in past year	1.24	0.91	1.68	1.29	0.93	1.79	**1.79**	1.13	2.84
Self-rated health, fair or poor	**1.78**	1.16	2.71	1.12	0.67	1.89	1.31	0.72	2.40
Current alcohol drinker	0.95	0.71	1.27	1.07	0.78	1.47	0.80	0.50	1.26
Depressive symptoms, (CES-D > 16)	0.70	0.29	1.67	0.75	0.30	1.92	**2.49**	1.05	5.91
Grip Strength (per SD)	1.15	0.94	1.41	0.87	0.68	1.10	0.91	0.64	1.28
Biomarkers									
Hemoglobin (per SD)	0.97	0.83	1.14	0.92	0.78	1.09	0.92	0.72	1.17
Serum Albumin (per SD)	**0.81**	0.70	0.93	**0.85**	0.72	0.99	0.87	0.70	1.09
Serum Vitamin D, Deficient (<20 ng/ml)	0.97	0.70	1.36	1.38	0.96	1.97	0.72	0.43	1.21
ApoE-e4+	**1.44**	1.06	1.95	0.72	0.50	1.05	**2.09**	1.33	3.28
Other									
DSST (per SD)	**0.57**	0.48	0.68	0.84	0.70	1.01	**0.47**	0.36	0.62
400 m gait speed (per SD)	**0.81**	0.68	0.96	**0.67**	0.56	0.81	**0.49**	0.37	0.64
*Sensitivity analysis*									
*CES-D > 10	1.00	0.62	1.62	0.94	0.56	1.57	1.58	0.85	2.95

SD = Standard Deviation, Depressive symptoms = score of > 16 on the Center for Epidemiologic Studies Depression Scale (CES-D-20), APOE-4+ = Apolipoprotein e4+, DSST = Digit Symbol Substitution Test. Bold text means significant value.

## 4. Discussion

Across the four decline categories, different patterns of risk factors emerged. Having less than a high school education and poor self-rated health were significantly related to higher odds of cognitive decline but were not related to physical decline or dual decline. Losing weight and depressive symptoms were significant risk factors for dual decline, but not related to the other categories. *APOE*-ε4 and DSST were significant predictors of cognitive decline and dual decline, and 400 m walk was significant across all three groups.

Evidence from large epidemiological studies have consistently shown that educational attainment influences rates of cognitive decline and risk of dementia (Stern et al., 1994). The association between self-rated health and cognitive decline is more novel, although it can be postulated that self-report engages a mental representation of personal history that consists of semantic and episodic knowledge (Jylha, 2009). This may provide rationale that self-rated health has a cognitive underpinning which we were able to detect as a significant predictor of subsequent cognitive decline.

Our results also showed that *APOE*-ε4 was a significant risk factor for cognitive decline and dual decline. *APOE*-ε4 has been widely shown to be a significant risk factor for Alzheimer’s disease and dementia (Corder et al., 1993). Less is known about *APOE*-ε4 and the association with physical decline, however in a recent study, Stringa et al. (2020) examined the modulation of *APOE*-ε4 on cognition including an interaction with self-reported physical activity in three longitudinal cohort studies: Longitudinal Aging Study Amsterdam, InCHIANTI, and Rotterdam Study. *APOE*-ε4 carriers had higher odds of cognitive decline in these cohorts, although there was no significant interaction between self-reported physical activity, *APOE*-ε4, and cognitive decline. This supports our finding that *APOE*-ε4 increases the risk of cognitive decline, however we also found a significant association between *APOE*-ε4 and dual decline. It may be that the association between *APOE*–ε4 and dual decline was simply driven by the cognitive portion of dual decline. Physical function was measured objectively in the Health ABC study using the 400 m walk and we found that faster 400 m walk time was related to lower odds of cognitive, physical, and dual decline.

Depressive symptoms and weight loss were uniquely related to dual decline. Depressive symptoms increased the odds by nearly 2.5-fold that a person would develop dual decline. Major depressive disorder is a common mental health problem for older adults and has been correlated with increased risk of falls (Kvelde et al., 2013), slower gait (Brandler et al., 2012), and increased executive dysfunction (Koenig et al., 2014). A systematic review examining this “triad” of physical function decline, cognitive decline, and depression was supported by 12 out of 15 studies suggesting a linkage among these factors (Patience et al., 2019). The basis for this connection is not fully understood, but this may be an important area of research in the future. To note, the number of participants with depressive symptoms was 3% (49/115) which may limit the generalizability of our study. In the cognitive decline and physical decline groups, depressive symptoms was not significant although the OR’s appear to look protective. This could be due to fact that those with depressive symptoms tended to be grouped into dual decline as opposed to only a single decline. Depression represents a potent modifiable risk factor and more research is needed to understand the consequences associated with cognitive and physical decline.

The first study examining predictors of dual decline and using a four group trajectory model (Atkinson et al., 2005) found smoking (OR = 5.66, 95% CI 1.49–21.54) and low hemoglobin (OR 0.68, 95% CI 0.47–0.98) to be unique predictors of dual decline in older women from the Women’s Health and Aging Study. Our results did not confirm those results, but our sample was more diverse including both men and women, and different methods were used to construct our definition of dual decline.

Strengths of this study include a longitudinal, well-described sample of over 1,500 older adults. The key strengths of Health ABC are the in-depth health, physical function, and clinical examinations administered annually or bi-annually in a healthy, well-functioning sample of older adults. Our study used the SPPB, as opposed to gait speed alone, which provides more information about function and included measures of gait speed, chair stand, and balance. This is the first study to use this approach as well as 17 different predictors. We chose to use a prospective longitudinal approach because participants at baseline reported no difficulty with physical function or cognitive impairment, and in this presumably healthy sample we could evaluate subsequent decline.

Our study has limitations, particularly since it used secondary data. The baseline age range of 70–79 and a presumably healthy cohort (i.e., self-reported no difficulties in cognition or physical function at baseline) was criterion for the Health ABC Study. We acknowledge that using self-reported cognitive status as a criterion for inclusion or exclusion in Health ABC is a limitation, but concerns are mitigated by the fact that these participants were able to complete procedures and participate in the study for a minimum of 4 years. Another weakness is that our results only included those with complete data, therefore individuals with missing data were not captured. The group size, specifically for dual decline, was slightly underpowered to detect meaningful differences. Since the study was exploratory in nature, we believe that our results contribute to an important emerging topic and warrants replication in a larger sample. The use of a global measure of cognition, the 3MSE, is a weakness because it is a single general test of cognition and not as comprehensive as a full neuropsychological battery. Also, predictors such as weight loss, depression, and alcohol drinking were from one visit at baseline, and we are unable to determine if these conditions were acute vs. chronic/habitual. In the future, it would be interesting to compare short term vs. long term predictors of decline.

More research is needed to further explore the mechanisms and the connection between cognitive health and physical health. Many have posited the role of the central nervous system and the hippocampus as being an important contributor to decline (Sorond et al., 2015). Of note, imaging measures were not available on our sample of participants. However, given the role of psychosocial factors (i.e., depression, walking speed) these factors are more easily measurable and can provide valuable information about a person’s cognitive and physical function.

## 5. Conclusion

Our study provides a longitudinal assessment of cognitive, physical, and dual decline among older adults providing new evidence for risk factors of decline. Future research should examine the role of psychosocial factors as they relate to cognitive and physical function and specifically target modifiable factors which may help reduce the burden of cognitive and physical decline among older adults.

## Data availability statement

Publicly available datasets were analyzed in this study. This data can be found at: https://healthabc.nia.nih.gov/.

## Author contributions

EH, SK, KH, and XL: conceptualization, methodology, and editing manuscript. EH: writing. SK and EH: funding acquisition. All authors contributed to the article and approved the submitted version.

## Funding

This research was supported by the National Institutes of Health/ National Institute on Aging (F32 AG058457) and the Wake Forest Claude D. Pepper Center (P30AG021332).

## Conflict of interest

The authors declare that the research was conducted in the absence of any commercial or financial relationships that could be construed as a potential conflict of interest.

## Publisher’s note

All claims expressed in this article are solely those of the authors and do not necessarily represent those of their affiliated organizations, or those of the publisher, the editors and the reviewers. Any product that may be evaluated in this article, or claim that may be made by its manufacturer, is not guaranteed or endorsed by the publisher.
